# P01 Urinary tract infections caused by ESBL-producing *Escherichia coli* in outpatients from Sarajevo, Bosnia and Herzegovina

**DOI:** 10.1093/jacamr/dlac053.001

**Published:** 2022-05-31

**Authors:** Sejla Kotoric Keser, Amina Obradovic Balihodzic

**Affiliations:** 1 Clinical University Center Sarajevo, Bosnia and Herzegovina; 2 Institute for Public Health Sarajevo, Bosnia and Herzegovina

## Abstract

**Background:**

Urinary tract infections (UTIs) are common infections acquired in the community. Knowledge of local antibiotic susceptibility profile is important in decision-making of the optimal choice for empirical antimicrobial therapy and its duration. ESBL-producing strains of Enterobacterales are considered to be emerging pathogens. Recurrent UTI is one of the risk factors for infection with ESBL-producing bacteria.

**Objectives:**

To report the prevalence of *Escherichia coli* uropathogens and the frequency of ESBL-producing *E. coli* strains isolated from urine of outpatients in Sarajevo Canton in Bosnia and Herzegovina.

**Materials and methods:**

In this retrospective study, we collected antibiotic susceptibility data from urine samples analysed in the Microbiology Laboratory of the Institute for Public Health of Canton Sarajevo from 1 January to 31 December 2021. Urine samples were inoculated on CUA III Liofilchem® medium and incubated at 37°C for 24–48 h. A count of ≥ 100 000 cfu/mL of urine was considered positive after incubation, and these isolates were identified. The antimicrobial susceptibility testing was performed using the disc diffusion method, according to EUCAST guidelines and results interpreted following EUCAST breakpoint tables v.10.

**Results:**

There were 34 087 urine samples from outpatients analysed in 2021. Out of this number, 23.72% (*n* = 8085) were positive. *E. coli* was identified in 70.27% (*n* = 5682) samples. ESBL *E. coli* was identified in 3.36% (*n* = 191) of all *E. coli* positive samples.

**Conclusions:**

Geographic variations in pathogen occurrence and susceptibility profiles require continuous monitoring to provide information to guide the empirical therapeutic options.

